# *ALDH2* genotype modulates the association between alcohol consumption and AST/ALT ratio among middle-aged Japanese men: a genome-wide G × E interaction analysis

**DOI:** 10.1038/s41598-020-73263-1

**Published:** 2020-10-01

**Authors:** Yoichi Sutoh, Tsuyoshi Hachiya, Yuji Suzuki, Shohei Komaki, Hideki Ohmomo, Keisuke Kakisaka, Ting Wang, Yasuhiro Takikawa, Atsushi Shimizu

**Affiliations:** 1 Division of Biomedical Information Analysis, Iwate Tohoku Medical Megabank Organization, Disaster Reconstruction Center, Iwate Medical University, 1-1-1 Idaidori, Yahaba, Iwate 028-3694 Japan; 2 Division of Hepatology, Department of Internal Medicine, Iwate Medical University, Yahaba, Japan; 3 Division of Biomedical Research and Development, Institute of Biomedical Sciences, Iwate Medical University, Morioka, Iwate Japan; 4 Division of Biomedical Information Analysis, Institute for Biomedical Sciences, Iwate Medical University, 1-1-1 Idaidori, Yahaba, Iwate 028-3694 Japan

**Keywords:** Genome-wide association studies, Diagnostic markers, Genetics research, Alcoholic liver disease, Risk factors

## Abstract

Liver tests (LT), especially to measure AST, ALT and GGT levels, are widely used to evaluate the risk of alcohol-related liver disease (ALD). In this study, we investigated the potential genetic factors that modulate the association between LTs and alcohol consumption. We conducted a genome-wide interaction meta-analysis in 7856 Japanese subjects from Tohoku Medical Megabank Community-Based Cohort (TMM CommCohort) study recruited in 2013, and identified 2 loci (12q24 and 2p16) with genome-wide significance (*P* > 5 × 10^–8^). The significant variants in the 12q24 included rs671, a variant associated with alcohol intolerance and located at a coding exon of *ALDH2*. We found that the amount of alcohol consumption was associated with increased level AST/ALT ratio among the subjects with the rs671 GA genotype. The elevated AST/ALT ratio among subjects with moderate-to-high levels of drinking behavior and the rs671 GA genotype was due to decreased levels of ALT, which was not accompanied with significant differences in AST levels. Although the interaction effect was significant in both men and women, the effect was much larger in men. Our results suggest that the impact of alcohol consumption on LT varies according to the *ALDH2* genotype, providing an insight for the accurate screening of ALD in drinkers with the rs671 GA genotype.

## Introduction

Alcohol-related liver disease (ALD) is a common medical complication caused by excessive alcohol consumption and comprises around 50% of the global burden of liver disease^1–4^. The most widely recognized forms of ALD are simple steatosis, alcoholic hepatitis, alcohol-related cirrhosis, and hepatocellular carcinoma^1,5,6^. Aspartate aminotransferase (AST), alanine aminotransferase (ALT) and gamma-glutamyl transpeptidase (GGT) are the major targets of liver tests (LT), which exhibit increase levels in serum of patients with ALD. The serum AST levels in patients with ALD tend to be higher than serum ALT levels. An AST/ALT ratio over 2.0 is a key indicator of ALD^7–9^.

Although LT are surrogate markers of ALD, the concentrations of liver enzymes in the serum are influenced other factors, including age, sex, and body mass index (BMI), as well as genetic factors^10–15^. In particular, genetic factors have a considerable impact on LT; a study on 5380 pairs of twins from the Twins UK registry estimated a (narrow-sense) heritability of ALT, AST, and GGT of 32%, 40%, and 69%, respectively^15^; similarly, a study on 3,375 pairs of twins from the Australian Twin Registry estimated the heritability of ALT, AST, and GGT to be 48%, 32%, and 52%, respectively^16^. Additionally, multiple studies suggested that the risk of alcohol misuse have heritability, although the scale is still controversial^17^. These genetic risks of alcohol misuse may ultimately result in increases in LT levels. Therefore, the heritability in LT seems to include the influence of genetic factors affecting LT via the mediation of dietary habits, such as alcohol consumption^18^. The influence of in the genetic factor via human behaviour potentially have some impact on the accuracy of screening for ALD.

A recent genome-wide association study (GWAS) studied 162,255 Japanese individuals and identified 27, 25, and 42 variants associated with ALT, AST, and GGT, respectively^19^. The sum of the contribution to heritability by the identified variants were estimated to be 1.34%, 1.31%, and 6.12% for ALT, AST, and GGT, respectively. However, in the context of the identification of the influence of genetic background on ALD screening, these previous estimates based on the general population cannot be applied for the effects of genetic factors on LT in drinkers. As ALD is a disease that depends on alcohol intake, conditional genetic effects in the drinkers, such as the interaction effect between genetic variants and the amount of alcohol consumption, is clinically important to understand the influence of genetic background on ALD screening.

The recently developed genome-wide variant × environment interaction analysis is a promising approach for the identification of genetic factors associated with markers indicative of risk for lifestyle diseases, via an interaction effect with environmental factor^20^. A previous study used genome-wide interaction analysis, resulting in the successful identification of the interaction between novel variants and the amount of daily sodium intakes in blood pressure^21^.

In this study, we report on the first population-based genome-wide interaction analysis used to identify genetic factors which influence LT in terms of daily alcohol consumption using a total 7856 Japanese individuals, comprised of residents from two prefectures. Using meta-analysis of the summaries of the genome-wide interaction analysis, we found that a variant in aldehyde dehydrogenase 2 (*ALDH2*) was significantly associated with ALT levels and the AST/ALT ratio through moderate-to-high alcohol consumption.

## Methods

### Study subjects

The Tohoku Medical Megabank Community-Based Cohort (TMM CommCohort) study was designed and as previously described^22^. Briefly, 20–75-year-old residents from Iwate and Miyagi, which are the Pacific coast prefectures in Northeast Japan, were recruited between May 2013 and March 2016. To control for unmeasured biases, individuals from Miyagi and Iwate were treated as separate sub-cohorts.

Physiological, urine, and blood tests were conducted at the time of enrolment. The levels of GGT, AST, and ALT were measured using standardized clinical laboratory techniques based on the standard protocol of the Japan Society of Clinical Chemistry (JSCC)^23^.

The medical history and lifestyles, including drinking habits, of the enrolled subjects were documented using self-administered questionnaires. In the questionnaires, current drinking status was defined in four categories: “current drinker (drinking more than once in a month)”, “former drinker”, “never (or almost never) drinker”, and “never drinker because of his/her predisposition to rejecting alcohol.” In this study, we treated only “current drinker” as a drinker, and others (i.e. “former drinker,” “never (or almost never) drinker,” and “never drinker because of his/her predisposition to rejecting alcohol”) as a non-drinker. Drinking frequency (drinking opportunity in a week) was reported by 6 categories: “less than 1 day/month”, “1–3 days/month”, “1–2 days/week”, “3–4 days/week”, “5–6 days/week”, and “every day.” We converted the answers into numeric values: 0, 0.5, 1.5, 3.5, 5.5, and 7 days/week, respectively. Weekly alcohol consumption (WAC) was denoted as the sum of ethanol content (g) for each type of beverages drunk in a week. The ethanol content of each type of alcoholic beverage was considered as follows: 180 ml sake (rice wine) as 23 g, 180 ml shochu (white spirits) as 36 g, 180 ml of chu-hai (cocktail using shochu) as 12.96 g, 633 ml beer as 23 g, 30 ml whisky as 10 g, and 100 ml wine as 12 g^24^. The daily alcohol consumption (DAC) was calculated by dividing WAC by 7 days. The subjects were stratified by DAC into 5 tiers, based on standard US drinks (14 g alcohol)^25^: tier 0 (DAC (drinks/day) < 0.1); tier 1 (0.1 ≤ DAC < 1); tier 2 (1 ≤ DAC < 2); tier 3 (2 ≤ DAC < 3); tier 4 (3 ≤ DAC). We defined the alcohol consumption for non-drinkers as 0.

The study was approved by the Institutional Review Board of Iwate Medical University and Tohoku University. All participants provided written informed consent. This study was conducted according to the principles expressed in the Declaration of Helsinki.

### Genotyping and genotype imputation

The procedure of genotyping and genotype imputation was performed as previously described^21,26,27^. Briefly, 9966 participants in the TMM CommCohort study, enrolled in 2013, were genotyped using a HumanOmniExpressExome BeadChip Array (Illumina Inc., San Diego, CA, USA). Subjects compatible with the following criteria were excluded from analysis: low call rate (< 0.99), sex-mismatch between questionnaire and genotype data, non-Japanese ancestry, or one of a close kinship pair (PI_HAT > 0.1875). The imputation of information on sex and the identification of close kinship pairs were conducted using the PLINK version 1.90b5.3. Variants with a low call rate (< 0.95), low Hardy–Weinberg equilibrium exact test *P*-value (*P* < 1 × 10^–6^), or low minor allele frequency (MAF; < 0.01) were also excluded. As a result, 1,127 individuals were removed, and 8839 subjects and 594,037 autosomal variants remained. After phasing by the SHAPEIT^28^ version 2.r900, imputation was conducted by Minimac3^29^ version 2.0.1 using the 1,000 Genomes reference panel phase 3^30^ as a reference. Variants with low-imputation quality (*R*^2^ < 0.8) were excluded. Finally, the remaining 7,129,678 variants were applied for subsequent analyses.

### Genome-wide interaction analysis and meta-analysis

Subjects who did not provide information on BMI, age, sex, alcohol consumption, or LT, such as AST, ALT, and GGT levels, were excluded. Additionally, subjects who had LT levels outside a range between a mean ± fourfold of standard deviation (SD), or who had a liver illness, such as hepatitis B, hepatitis C, liver cancer, or fatty liver disease, were also excluded. As a result, 983 individuals were excluded, and 7856 individuals remained. To perform a linear regression, GGT, AST, and ALT were log-transformed.

We performed polymorphism × environment interaction analysis in the enrolled Miyagi and Iwate residents, respectively. The method of the interaction analysis was performed as previously described^21^. Briefly, we fitted a linear regression model using a null hypothesis (H_0_), which lacked an interaction term, and an alternative hypothesis (H1), including interaction term, as follow:

$${\text{H}}0:{\text{ Y }} = \, \beta_{0} + \, \beta_{{\text{G}}} {\text{G }} + \, \beta_{{\text{E}}} {\text{E,}}$$

$${\text{H1}}:{\text{ Y }} = \, \beta_{0} + \, \beta_{{\text{G}}} {\text{G }} + \, \beta_{{\text{E}}} {\text{E }} + \, \beta_{{{\text{GE}}}} {\text{G}} \times {\text{E,}}$$

where Y is LT (AST/ALT ratio, or log-transformed GGT, AST or ALT), G is genotype variable, E is DAC (g/day) variable, β_0_ is the intercept, β_G_ is the coefficient for variable G, β_E_ is the coefficient for variable E, and β_GE_ is the coefficient for the interaction between G and E. The interaction analysis was adjusted for age, sex, BMI, and population structure in the genotype dataset (top 5 principal components [PCs] calculated using the PLINK software). The significance of the interaction term (β_GE_) was evaluated using the 1 *df* likelihood ratio test^20^.

The summaries of genome-wide interaction analysis for each prefectural population were applied for inverse-variant based meta-analysis using METAL (released on 2011-03-25)^31^. After genomic control correction, variants with *P*_*meta*_ < 5 × 10^–8^ were considered as genome-wide significant.

### Replication analysis

For our replication study, we used the pre-imputed dataset released by the TMM^32,33^. Within this dataset, we used the subsets genotyped using Omni2.5 SNP array (Illumina Inc., San Diego, CA, USA) as well as the customized genotyping array designed by the TMM based on the Axiom platform (Thermo Fisher Scientific, Waltham, MA USA), denoted as Japonica array version 2 (JPAv2). The genotyped data were pre-phased using SHAPEIT version 2 r837 and imputed using IMPUTE2 version 2.2.2 and 2KJPN with an allele frequency panel of ~ 2000 Japanese individuals^34–36^. After conducting the same quality control with the main dataset, 4,935,024 and 5,686,147 variants in the JPAv2 and Omni2.5 datasets, respectively, were selected for further analysis.

Replication analysis was conducted using the same exclusion criteria as those for the main analysis. Additionally, we excluded the Miyagi population in the subjects genotyped by JPAv2, because of small sample size (n = 678). The all subjects in the dataset genotyped by Omni2.5 belonged in the Miyagi population. Ultimately, 2791 and 1597 individuals for the JPAv2 and Omni2.5 dataset, respectively, were selected for replication analysis.

### Power calculation

The power calculation was conducted as previously reported^21^. Briefly, we assumed that residuals of age-, sex-, and BMI-adjusted LT were distributed according to the following genetic model: LT = β_E_E + β_G×E_ G × E, where variable E (alcohol consumption) was sampled from a normal distribution and variable G (genotype) was sampled according to assumed minor allele frequency (20% or 50%). The model parameters (β_E_) were estimated from the dataset used in the present study. β_G×E_ was assumed to be 0.25- to 2.5-fold β_E_. We simulated data for E and G for the Iwate and Miyagi populations, performed inverse-variance weighted meta-analysis, and recorded whether the interaction term achieved suggestive significance. This process was repeated for 1000 iterations to calculate the power of each parameter set.

### Estimation of genetic correlation and LD score regression intercept

We estimated the genetic correlation and LD score regression intercept using LDSC version 1.0.1^37,38^ and pre-computed LD scores for East Asians provided by the program developer. The LD score regression intercept was calculated using summary statistics. To estimate the genetic correlation between the LT traits, summary data from the published GWAS conducted in Japanese population were used^19^.

### Statistical analysis for polymorphism × environment interaction

The statistical analysis for the identified variants was conducted using R (version 3.5.1). To determine the trend in quantitative traits, the Junckheere-Terpstra test was performed using clinfun (version 1.0.15). To calculate the adjusted LT, we added the mean LT to the residual in the linear regression adjusted by age, sex, BMI and genotype, as described in a previous study^21^.

### Data availability

The datasets analyzed in the current study are not publicly available for ethical reasons. However, they can be made available upon request after approval from the Ethical Committee of Iwate Medical University, the Ethical Committee of Tohoku University, and the Materials and Information Distribution Review Committee of the TMM Project.

## Results

### Genome-wide interaction analyses

The characteristics of the study populations are shown in Table 1. The Miyagi residents were slightly younger and had a higher proportion of females than the Iwate residents. Moreover, the Miyagi residents had a slightly higher proportion of current drinkers than the Iwate residents, although the both groups had similar averages in terms of drinking frequency and alcohol consumption. The BMI, GGT, AST, ALT, and AST/ALT ratios were similar in the both groups.

**Table 1:** Characteristics of study populations.

	Iwate	Miyagi
*N*	3878	3978
Female, %	64.0	68.3
Age, year (mean ± SD)	63.1 ± 9.9	59.5 ± 11.9
BMI, kg/m^2^ (mean ± SD)	23.4 ± 3.4	23.4 ± 3.5
Current drinker, %	44.2	48.0
Drinking frequency, days/week (mean ± SD)	1.9 ± 2.8	1.9 ± 2.7
Alcohol consumption, g/day (mean ± SD)	11.9 ± 23.2	10.5 ± 21.1
AST, IU/L (mean ± SD)	36.6 ± 50.5	33.4 ± 43.6
ALT, IU/L (mean ± SD)	25.5 ± 11.2	24.1 ± 13.4
GGT, IU/L (mean ± SD)	22.1 ± 14.3	22.4 ± 18.0
AST/ALT (mean ± SD)	1.3 ± 0.4	1.2 ± 0.4

*BMI* body mass index, *AST* aspartate aminotransferase, *ALT* alanine aminotransferase, *GGT* γ-glutamyl transferase.

A significant genetic correlation was found between the LT traits calculated using summary data from the published GWAS in Japanese population (Supplementary Table S1). The power calculation of our meta-analysis indicated that the power reached ≥ 80% when a variant (MAF = 0.5) had an interaction effect of ~ 2.5-, ~ 1-, or ~ 0.5-fold greater than the size of the alcohol consumption effect (1 g/day) (Supplementary Table S2).

We performed meta-analysis using the summaries of the genome-wide interaction analysis in each prefectural population (Supplementary Fig. 1). The inflation factor (λ) of the observed test statistics against the expected test statistics in the meta-analysis was as follows: 1.117 [1.114–1.119] for the AST/ALT ratio (values in brackets indicates 95% confidence interval), 1.188 [1.186–1.190] for ALT, 1.490 [1.487–1.493] for AST, and 1.852 [1.848–1.855] for GGT. The ratio of the LD score intercept and the mean *χ*^2^ suggested that most of the inflation in the present analysis was caused by factors other than polygenic heritability (Supplementary Table S3)^37,38^. We conducted a genomic-control correction to control for the inflation of the test statistics^39^, and confirmed that the inflation was suppressed to residual levels.

**Figure 1:**
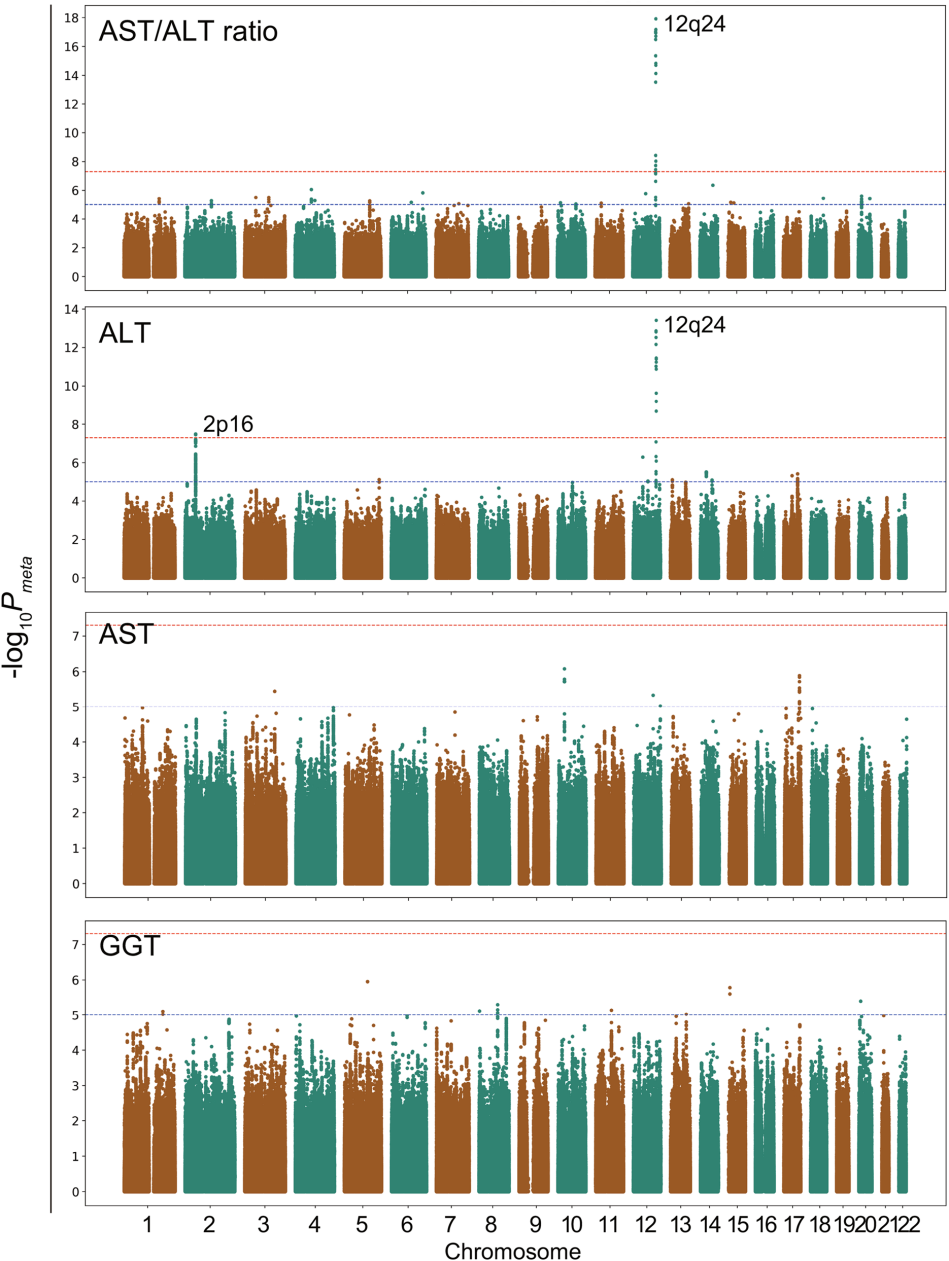
Genome-wide interaction meta-analysis for polymorphism × alcohol consumption interaction associating with LT. Summary of the genome-wide interaction meta-analysis for liver tests (LT) is shown as a Manhattan-plot. In the meta-analysis for AST/ALT ratio, a signal appeared in the 12q24 locus (the top figure). In the analysis for ALT, 2 signals were found in 12q24 and 2p16 (the 2nd figure from top). The y-axis indicates the negative log-transformed *P*-value for polymorphism × alcohol consumption interaction, and the x-axis indicates the positions of variants in reference genome (GRCh37/hg19), grouped by each chromosome. The observed test statistics were corrected by the genomic control. The red and blue horizonal lines indicate levels of genome-wide significance (*P* = 5 × 10^–8^) and suggestive significance (*P* = 1 × 10^–5^), respectively. *AST* aspartate aminotransferase, *ALT* alanine aminotransferase, *GGT* γ-glutamyl transferase.

**Figure 2:**
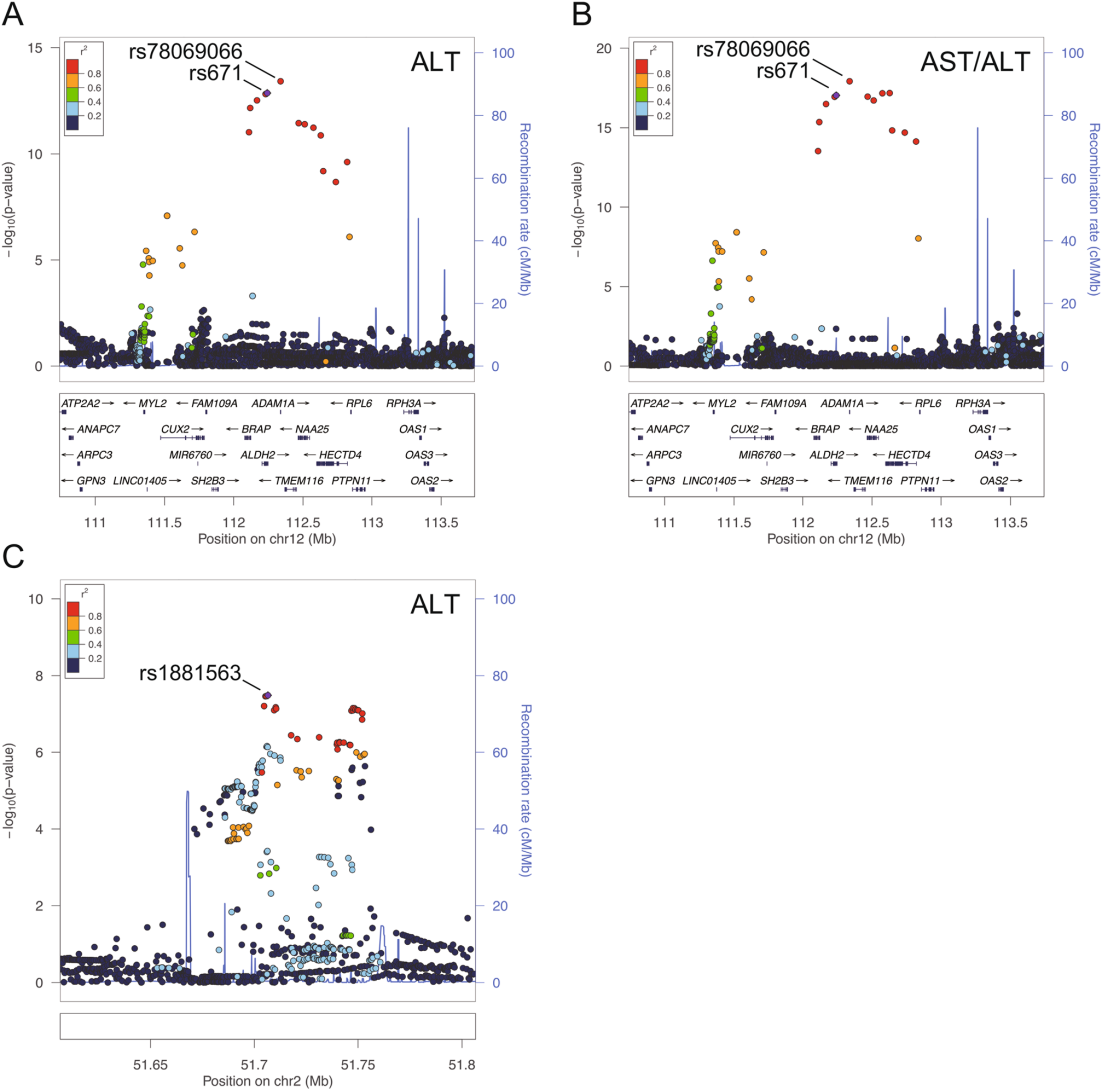
Variants around identified signals in the meta-analysis. Summary of the genome-wide interaction meta-analysis for LT represented by Locuszoom^64^, focusing on the variants identified around 12q24 (**A**,**B**) and 2p16 (**C**). The y-axis indicates the negative log-transformed *P*-value for variants × alcohol consumption interactions. The x-axis indicates the position of variants in the reference genome. The colors of the dots indicate the *R*^2^ values for the linkage disequilibrium (LD) in the present study. The reference variants for LD calculation were rs671 and rs1881563 in 12q24 and 2p16, respectively, which are shown in light purple. *AST* aspartate aminotransferase, *ALT* alanine aminotransferase.

The genomic-controlled summary of the meta-analysis was represented in the form of a Manhattan-plot (Fig. 1). We found that 16 and 17 interactions reached genome-wide significance (*P* < 5 × 10^–8^) in the meta-analysis for ALT and AST/ALT ratio, respectively (Table 2 and Supplementary Tables S4 and S5). Of those, 13 variants for the ALT and 17 variants for the AST/ALT ratio were localized on 12q24. Interestingly, all of the variants identified in 12q24 showed a moderate to strong linkage disequilibrium (LD) with rs671 (*R*^2^ > 0.62 in the present study) (Fig. 2A,B), which is the variant responsible for acute alcohol flashing, frequently found in East-Asian individuals^40^. Three significant ALT variants were identified in the locus of non-coding RNA (LOC730100), which is located on 2p16 (Fig. 2C and Supplementary Table S1). No variant reached a level of genome-wide significance in the meta-analysis for GGT and AST (Fig. 1).

**Table 2:** The top signals in the genome-wide meta-analysis for genotype × alcohol consumption interaction associating with LT.

rsID	Chr	Position^a^	Ref.	Alt	MAF	LT	*β*	SE (*β*)	*I*^2b^	*P*_*het*_^b^	*P*_*meta*_^c^
rs78069066	12	112,337,924	G	A	0.18	ALT	− 0.005	0.001	86.1	0.007	3.8 × 10^–14^
						AST/ALT	0.005	0.001	86.3	0.007	1.2 × 10^–18^
rs1881563	2	51,706,555	A	T	0.34	ALT	0.002	3.0 × 10^–4^	0	0.954	3.3 × 10^–8^

*Chr* chromosome number, *Ref* reference allele, *Alt* alternative allele, *MAF* minor allele frequency, *SE* standard error, *LT* liver test, *AST* aspartate aminotransferase, *ALT* alanine aminotransferase.

^a^Chromosomal position according to GRCh37/hg19 assembly.

^b^The *I*^2^ statistic and *P*-value for the heterogeneity statistic (*P*_*het*_) were calculated by METAL^31^.

^c^The *P*-value for the meta-analysis (*P*_*meta*_) was corrected by genomic control.

Next, we assessed the robustness of our analysis. Heritable covariates, such as BMI, in the analysis can lead biases, such as collider biases, in the main effect^41^. To assess the influence of biases on our results, we estimated the effect size and *P*-values of the identified variants without BMI (Supplementary Tables S6 and S7). In addition, we assessed the potential confounder in the genotype (G) × environment (E) interaction analysis by introducing terms for G × covariate interactions, namely G × BMI, G × age, and G × sex, as well as E × covariate interactions, namely E × BMI, E × age, and E × sex, into our analysis (Supplementary Tables S8 and S9)^42^. In the both cases, the effect size and *P*-values were found to shift slightly from the those in the main analysis.

Additionally, we evaluated the role of sex differences in the effects on the identified variants (Supplementary Tables S10–S12). The interaction effect of 12q24 (rs78069066) was found to be significant in men but not in women. In a meta-analysis using the results from both men and women, *P* for heterogeneity and *I*^2^ indicated that there was significant heterogeneity between the effect sizes between men and women. For example, for the AST/ALT ratio, *P*_*het*_ in the top signal of 12q24 (rs78069066) was 3.4 × 10^–3^ and 4.5 × 10^–3^ in the Iwate and Miyagi population, respectively. Furthermore, a significant level of heterogeneity was found in ALT for the Iwate population (*P*_*het*_ = 3.3 × 10^–4^) but not for the Miyagi population (*P*_*het*_ = 0.37). These results suggest that interaction effects were observed only among men.

Lastly, we conducted a replication analysis using the subgroups in the TMMCommCohort study, comprised of the independent subjects from the main analysis (Supplementary Tables S13–S15). The signal in 12q24 showed a significant *P*-value, which was lower than the Bonferroni threshold (For ALT, *P* = 0.05/2 loci; for AST/ALT ratio, *P* = 0.05**/**1 locus), while the signal in 2p16 showed no significant results in the replication study.

### Effect of genotype × environment interaction by stratified alcohol consumption

**Figure 3:**
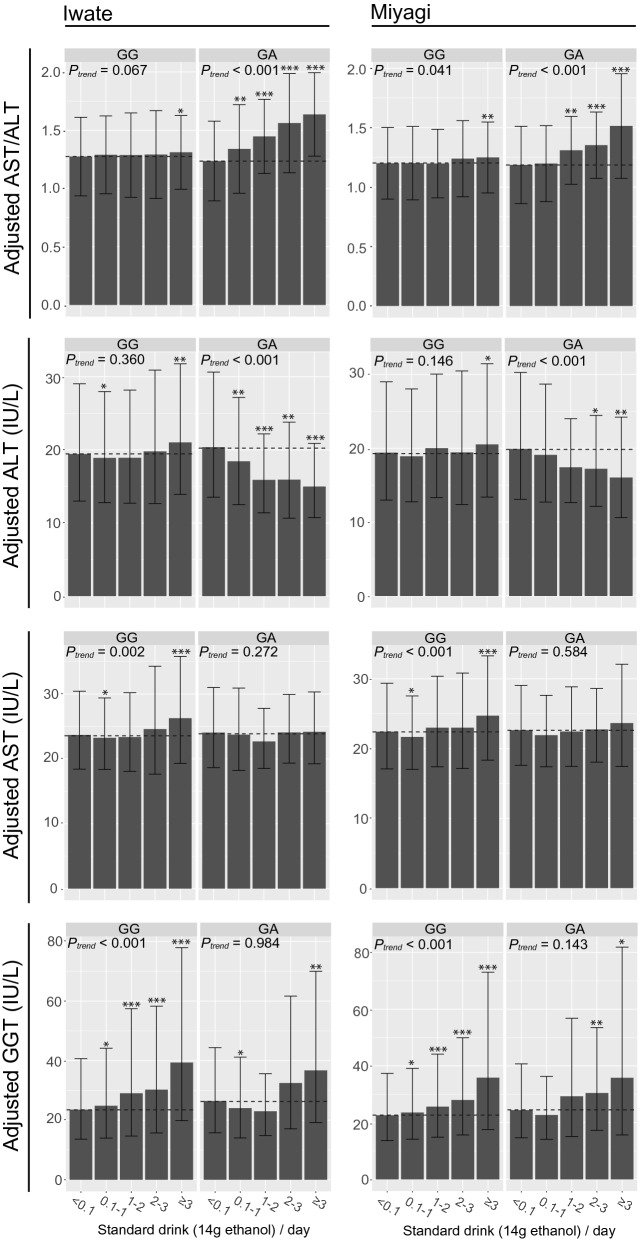
Adjusted LT stratified by alcohol consumption and rs671 genotype. The LT in the Iwate (left column) and Miyagi (right column) populations, adjusted by age, sex, BMI, and rs671 genotype, are represented as a bar plot. The population was stratified by rs671 genotypes, such as rs671 GG and GA, and 5 tiers of daily alcohol consumption (DAC), indicated by standard drink (14 g alcohol)/day. The rs671 AA carrier was excluded from analysis due to a small sample number. The *P*-value for trend (*P*_*trend*_) was estimated using the Jonckheere-Terpstra test. The horizonal dotted line indicates the value in < 0.1 drink/day. The adjusted LT was compared with LT in < 0.1 drink/day using Wilcoxon’s rank sum test. The *P*-values are represented as follows: ***P < 0.001; **P < 0.01; *P < 0.05. *AST* aspartate aminotransferase, *ALT* alanine aminotransferase, *GGT* γ-glutamyl transferase.

To evaluate the impact of the interaction between the genotype of the identified variant and alcohol consumption on LT, we stratified the subjects by genotype of the identified variant and alcohol consumption, then calculated the mean LT for each stratified group, adjusted for age, sex, and BMI, which are the known associating factors for LT^43,44^. Additionally, to exclude the effect of the genotype, which is independent of alcohol consumption, we used the genotype of the identified variant for the adjustment (Fig. 3 and Supplementary Fig. 2). Because the variants identified were highly linked each other, we used rs671, a variant known to affect the alcohol metabolism, as a representative variant of 12q24 in the subsequent analyses^40^. Note that we excluded the rs671 AA carrier from our analysis due to a small sample size (N = 119 in Iwate and N = 142 in Miyagi). We found that subjects who had the rs671 GA showed a clear increase in their AST/ALT ratio, according to drinking tiers (Fig. 3). In comparison with tier 0 (< 0.1 drinks/day), the AST/ALT ratio began to increase from tier 1 (0.1–1 drinks/day) in the Iwate population (*P* < 0.01), and tier 2 (1–2 drinks/day) in the Miyagi population (*P* < 0.01). In the rs671 GA carriers, the average of the adjusted AST/ALT ratio on tier 3 (2–3 drinks/day), middle-to-high alcohol consumption, reached up to ~ 1.6 in the Iwate population, indicating a ~ 25% increase from that on the tier 0. This increase in the adjusted AST/ALT ratio was clearly consistent with the ALT drop in the rs671 GA carriers, which is accompanied with an increased alcohol consumption; the adjusted ALT began to decreasing on tier 1 in the Iwate population (*P* < 0.01) and tier 3 in the Miyagi population (*P* < 0.05). In the Iwate population, the adjusted ALT on tier 3 decreased up to ~ 20% of that on tier 0. Additionally, the adjusted AST in the rs671 GA carriers showed no trend for any direction, according to the level of alcohol consumption. These results suggest that the increase in the AST/ALT ratio in rs671 GA carriers mainly depends on the ALT drop, according to increasing alcohol consumption, rather than an increase in AST.

Moreover, the decrease in ALT did not appear in the rs671 GG carriers in the corresponding levels of alcohol consumption. In comparison with tier 0, the adjusted AST/ALT ratio in the rs671 GG carriers showed no differences in any tiers, except tier 4 (≥ 3 drinks/day) (*P* < 0.05). In contrast to the rs671 GA carriers, the adjusted AST in the rs671 GG carriers indicated a significantly increasing trend (*P*_*trend*_ < 0.002). These results suggest that, the AST/ALT elevation in the rs671 GG carriers, which was observed in tier 4 of the Miyagi and Iwate populations, mainly resulted an increased AST, rather than a decrease in ALT, as observed in the rs671 GA carriers. In the rs671 GG carriers, GGT increased with increasing alcohol consumption (*P*_*trend*_ < 0.001). In the rs671 GA carriers, GGT significantly increased in tier 4 (*P* ≤ 0.01) in Iwate individuals, and tier 3 (*P* ≤ 0.01) and 4 (*P* ≤ 0.05) in Miyagi individuals, although the trend was not significant.

We also assessed the effect of sex on the interaction effect (Supplementary Fig. 2). In men, a significant reduction in ALT was observed in both the Iwate (*P*_*trend*_ < 0.001) and Miyagi (*P*_*trend*_ = 0.008) populations, as well as a significant increase in the AST/ALT ratio (*P*_*trend*_ < 0.001; Iwate and the Miyagi populations), in correlation with an increasing level of alcohol consumption. By contrast, these effects were not observed in women.

Similarly, we analyzed the interaction effect in rs1881563, the top signal in 2p16 (Supplementary Fig. 3). In contrast to rs671, rs1881563 showed no significant trend as alcohol consumption increased, except for rs1881563 AA carriers in the Iwate population (*P*_*trend*_ < 0.001). The rs1881563 TT carriers with heavy alcohol drinking (tier 4; ≥ 3 drinks/day) showed a significant increase in the adjusted ALT (P < 0.05 in the Iwate population and P < 0.01 in the Miyagi population).

## Discussion

In this study, we identified 2 loci associating LT with alcohol consumption. 12q24 is known as a locus harboring rs671, a missense variant of the *ALDH2* gene, which is responsible for acute alcohol flashing^40^. We stratified our analysis by alcohol consumption and the genotype of the GWAS identified variants and found a genotype-specific ALT drop in rs671 A allele holders under moderate-to-high alcohol consumption. To the best of our knowledge, the impact of the genotype × alcohol consumption interaction on LT has not been previously reported.

While AST is abundantly present in many different types of tissue in addition to the liver, such as skeletal, cardiac, and smooth muscle, ALT is present at low concentrations in non-hepatic tissues^45^. Therefore, the serum ALT levels are considered a more specific marker of liver injury, and the AST/ALT ratio is globally used as an indispensable markers in the diagnosis of ALD^46^. The decrease in genotype-specific ALT suggests that distinguishing the upper limit of normal (ULN) ALT levels by the ALDH2 genotype is necessary to prevent the underestimation of health risks in heavy drinkers.

ALDH2 is a homo-tetramer enzyme for the nicotinamide adenine dinucleotide (NAD)-dependent oxidation of acetaldehyde and plays major role in the alcohol metabolism in the liver. An *ALDH2* allele, rs671 A, is an allele responsible for alcohol intolerance, which causes marked facial flushing and mild to moderate symptoms of intoxication^47,48^. rs671 is a non-synonymous G-to-A transition in an ALDH2 protein-coding region and causes glu504-to-lys mutation, which has a dominant negative effect on the entire enzyme, losing almost all of the activity in the homo-tetramer complex^49^. Even the incorporation of a single subunit derived from the rs671 A allele significantly reduced the overall activity of the ALDH2 tetramer^50^. Compared with the ALDH2 activity in rs671 GG carriers, the ALDH2 activity in rs671 GA carriers is ~ 6%, while that in rs671 AA carriers is negligible.

rs671 A is a common allele in East-Asian individuals; in the present study, the minor allele frequency (MAF) of rs671 was 0.18 (Supplementary Table S2). The association between the rs671 genotype and LT, including GGT, ALT, and AST, has been reported in several studies so far^51,52^. In addition, a previous study reported that the association between GGT and rs671 is dependent on drinking status (non-drinker/periodically drinker/everyday drinker)^51^. More recently, GWAS for ~ 14,700 Japanese individuals suggested that rs671 and rs3782886 were significantly associated with GGT (*P* = 4.5 × 10^–9^) and ALT (*P* = 5.5 × 10^–9^), respectively^53^. These previous studies, however, were conducted without an explicit distinction between the interaction effect from other effects, such as the simple effect of variants or alcohol consumption. Therefore, the present study is the first report on a genome-wide distribution and the impact of the variants on LT via genotype × alcohol consumption interactions, based on a community-based cohort study.

Drinkers with a rs671 A allele are relatively rare^51^, and previous meta-analyses have suggested that rs671 A allele has a protective effect on ALD, as well as dependence, due to inducing avoidance for drinking by discomfort resulting from an intolerance to alcohol^54^. On the other hand, the limited ALDH2 activity in the alcohol metabolism increases the risk of acetaldehyde exposure, which has been reported to be associated with several diseases, such as carcinogenesis and osteoporosis^55–57^. For example, in heavy drinkers of Japanese origin (~ 75 ml/day), rs671 GA carriers showed a greater risk of esophageal carcinogenesis than GG carriers (OR = 19.4 [4.67–80.8])^58^. Moreover, in rs671 GA carriers, drinkers who drink more than once a week and more than 50 g ethanol each time showed significantly higher levels of rectal cancer risk than non-drinkers (OR = 8.07 [1.88–34.7])^59^.

In the present study, we identified a genotype-specific response to alcohol consumption, accompanied by an increased AST/ALT ratio as a result of a reduction in ALT (Fig. 3). Generally, an increased AST/ALT ratio is observed in individuals with a chronic alcohol use disorder^9^, where this elevation is explained as follows: (1) acetaldehyde promotes the decay of pyridoxal 5′-phosphate, an activated form of vitamin B6 required for the activity of ALT and AST^60^; (2) alcohol stimulates the synthesis and release of mitochondrial AST; (3) as a result of a reduction in ALT activity and the upregulation of AST, the AST/ALT ratio increases, depending on the level of alcohol consumption. In a present study, however, no significant trend was observed for AST in rs671 GA carriers (*P*_*trend*_ = 0.272 in the Iwate population, and *P*_*trend*_ = 0.584 in the Miyagi population) (Fig. 3). Additionally, the lack of a significant response in AST suggests that the increase in the AST/ALT ratio does not result from a liver injury. Therefore, the genotype-specific increase in AST/ALT may be dependent on different physiological and pathological processes in individuals with a chronic alcohol use disorder and typical liver injuries. Further biomedical analysis will be needed to fully elucidate the mechanism of a genotype-specific reduction in ALT.

In the genome-wide interaction meta-analysis, we identified a novel association of variants which focused in an intron of non-coding RNA (LOC730100) located in 2p16, suggesting that the *LOC730100* locus was associated with ALT via interactions with alcohol consumption (Supplementary Fig. 2). We surveyed this locus in the GWAS catalog, however, no suggestive (*P* < 1 × 10^–5^) entry was found with regards to LT (Supplementary Table S16)^61^. Recently, a study suggested that *LOC730100* expression enhances the proliferation of glioma cells via the regulation of the miR-760/FOXA1 axis^62^. FOXA1 is a transcriptional activator for liver-specific transcripts^63^. This suggest that the up-regulation of *LOC730100* expression could affect liver function. However, there is currently a lack of studies on the role of *LOC730100* expression in the liver.

When the variants were assessed for potential biases (i.e. collider biases) and confounding (i.e. genotype × covariate and environment × covariate interactions), our results for the main genotype × environment interaction analysis were found to shift slightly. This indicates that the initial results of the interaction analysis may be biased. However, this did not appear to have a significant effect on our conclusions (Supplementary Tables S6–S9).

Our genotype × environment interaction analysis also indicated sex-based differences in the interaction effect between the male and female subgroups (Supplementary Table S10–S12). The 12q24 signal was replicated in the replication dataset, demonstrating the robustness of our results (Supplementary Table S13–S15). On the other hand, the 2p16 signal was not replicated in the dataset. Because a relatively smaller effect size was found for the 2p16 signal, further studies using a larger cohort will be needed to confirm these results.

This study contains some limitations. Because the variants identified in the 12q24 had a strong LD, we could not conclude whether rs671 is a true causal variant in the genotype × alcohol consumption interaction^54^. However, the functions of the rs671 A allele, which inhibits the alcohol metabolism, is sufficient to assume that it is the primary candidate of the causal variant. Future studies using another cohort of an East-Asian population will be needed to determine the causal variant. Additionally, the present results could be biased by several (unmeasured) factors, although we assessed several possibilities of the biases, including the collider bias. The future meta-analysis using multiple cohorts could be helpful to confirm the present results.

In summary, this genome-wide interaction study identified the significant interactions between genotypes and alcohol consumption, as a factor associated with the ALT levels and AST/ALT ratio. We found a genotype-specific response in ALT associated with an increased alcohol consumption. Since carriers of the rs671 A allele are rare, apart from in East-Asians populations, similar genome-wide interaction analysis in other populations, such as Africans and Europeans, will be needed to identify over- or underestimations of risk in the current ULN. These interaction analyses may provide insights into the accurate screening of ALD based on individual genetic backgrounds.

## Supplementary information

Supplementary Figures.

Supplementary Tables.
