# Supplementary material for: ALDH2 genotype modulates the association between alcohol consumption and AST/ALT ratio among middle-aged Japanese men: a genome-wide G × E interaction analysis
Source: Sci Rep. 2020 Oct 1;10:16227. doi: 10.1038/s41598-020-73263-1 (PMC7530747; doi:10.1038/s41598-020-73263-1)
Supplement: Supplementary file 1 — Supplementary Figures. [file 41598_2020_73263_MOESM1_ESM.docx]

**Supplementary Information**

***ALDH2* genotype modulates the association between alcohol consumption and AST/ALT ratio among middle-aged Japanese men: A genome-wide G×E interaction analysis**

**Yoichi Sutoh^1^, Tsuyoshi Hachiya^1^, Yuji Suzuki^2^, Shohei Komaki^1^, Hideki Ohmomo^1^, Keisuke Kakisaka^2^, Ting Wang^3^, Yasuhiro Takikawa^2^, Atsushi Shimizu^1,4*^**

^1^Division of Biomedical Information Analysis, Iwate Tohoku Medical Megabank Organization, Disaster Reconstruction Center, Iwate Medical University, Yahaba, Japan

^2^Division of Hepatology, Department of Internal Medicine, Iwate Medical University, Yahaba, Japan

^3^Division of Biomedical Research and Development, Institute of Biomedical Sciences, Iwate Medical University, Morioka, Iwate, Japan

^4^Biomedical Laboratory Sciences, Institute for Biomedical Sciences, Iwate Medical University, Yahaba, Japan


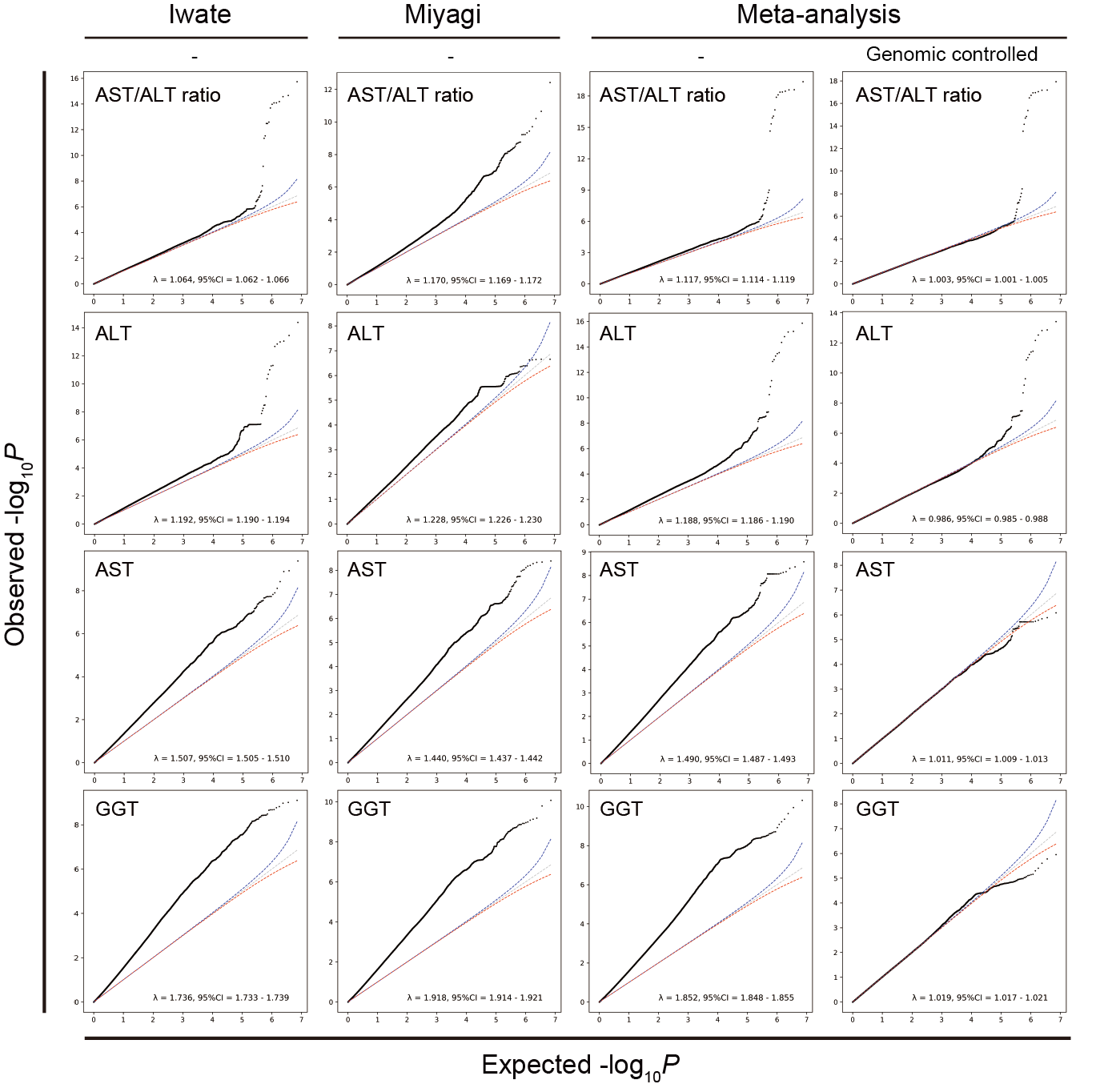


**Supplementary Figure 1. Quantile-quantile (qq) plot for polymorphism × alcohol consumption interaction analysis and meta-analysis.**

Summary of genome-wide interaction analysis and meta-analysis for liver function test (LT), represented in a quantile-quantile (qq) plot. The y-axis indicates the negative log-transformed observed *P*-value in the Iwate population (left), the Miyagi population (2nd column from the left) and their meta-analysis (3rd and 4th from the left); the x-axis indicates the expected distribution of *P*-values in null hypothesis. The observed *P*-values in the meta-analysis were corrected by genomic control (the 4th column from the left). The area between the red and blue line indicates the 95% confidence intervals. The genomic inflation factor (λ) of the observed *P*-value is shown in the plot with a 95% confidence interval (95%CI). AST, aspartate aminotransferase; ALT, alanine aminotransferase; GGT, γ-glutamyl transferase.

**
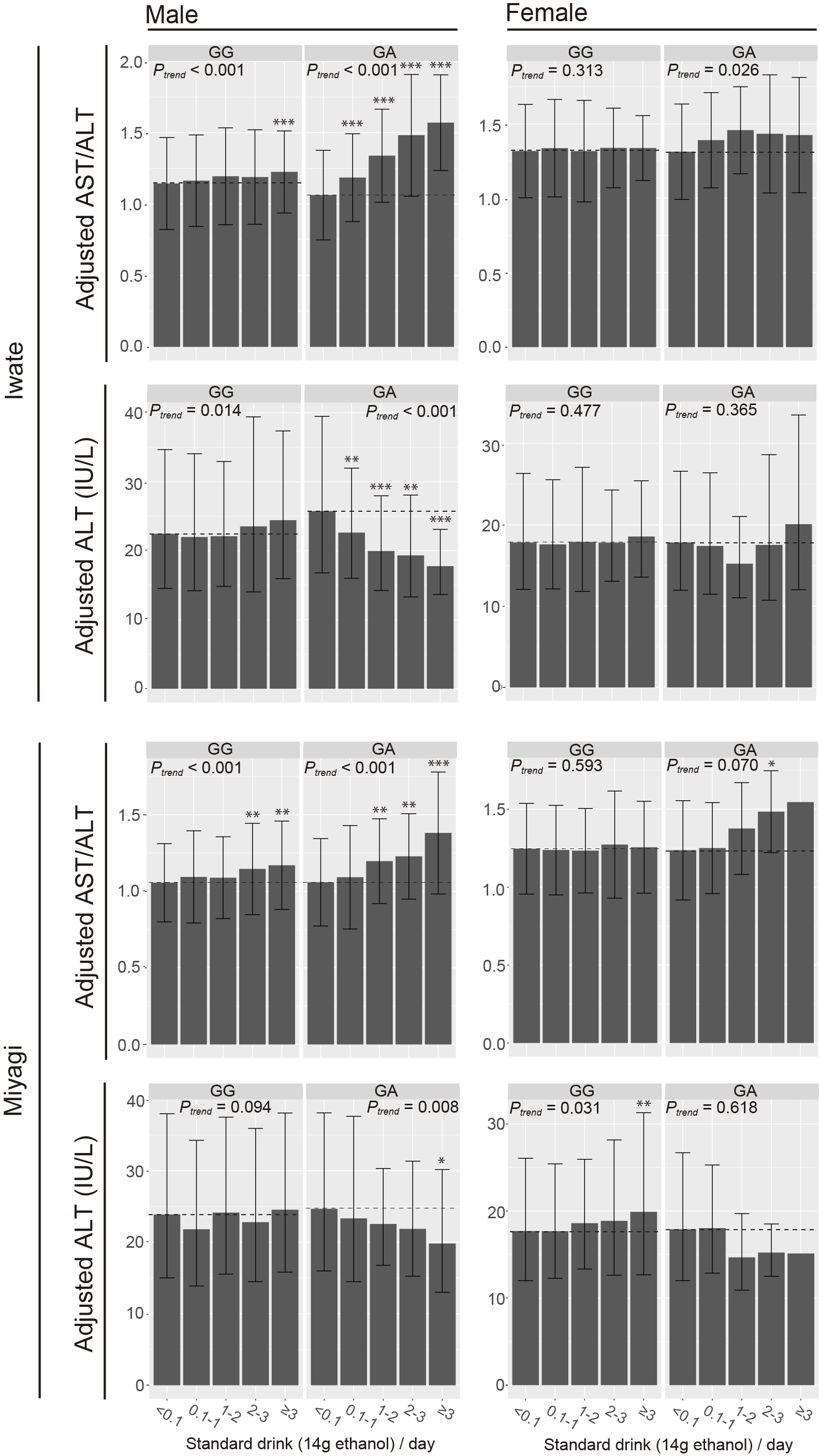
**

**Supplementary Figure 2. Sex differences in the interaction effect.**

The LT in the male (left column) and female (right column), adjusted by age, BMI, and rs1881563 genotype, represented in a bar plot. The population was stratified into 5 tiers based on daily alcohol consumption (DAC), indicated by standard drink (14g alcohol) / day. *P*-value for trend (*P_trend_*) was estimated using the Jonckheere-Terpstra test. The horizonal dotted line indicates the value in < 0.1 drink/day. The adjusted LT was compared with the LT in < 0.1 drink/day using Wilcoxon’s rank sum test. The *P*-values are presented as follows: ***, *P* < 0.001; **, *P* < 0.01; *, *P* < 0.05. ALT, alanine aminotransferase; AST, aspartate aminotransferase.


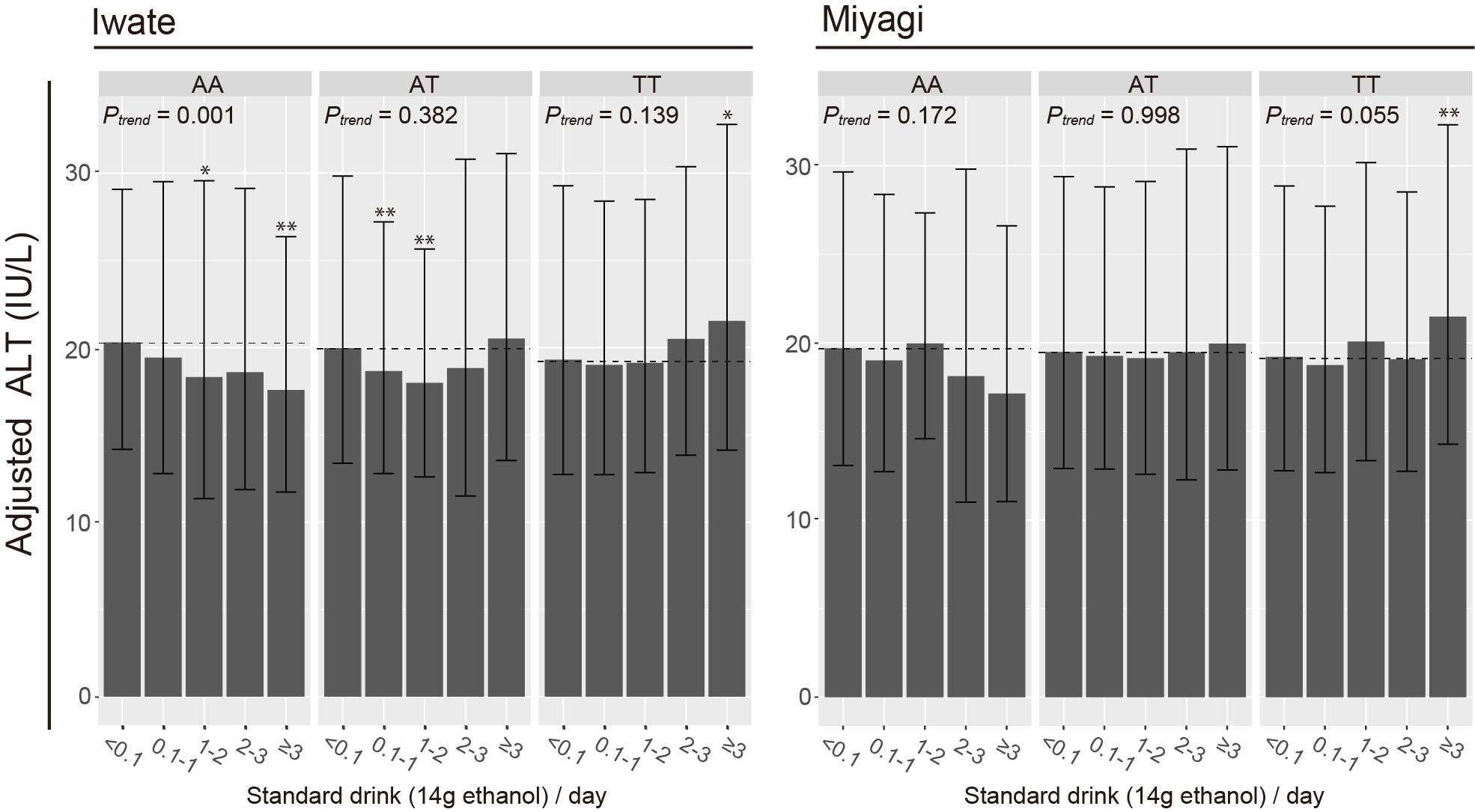


**Supplementary Figure 3. Adjusted LT stratified by alcohol consumption.**

The LT in the population of Iwate (left column) and Miyagi (right column), adjusted by age, sex, BMI, and rs1881563 genotype, represented in a bar plot. The population was stratified into 5 tiers based on daily alcohol consumption (DAC), indicated by standard drink (14g alcohol) / day. *P*-value for trend (*P_trend_*) was estimated using the Jonckheere-Terpstra test. The horizonal dotted line indicates the value in < 0.1 drink/day. The adjusted LT was compared with the LT in < 0.1 drink/day using Wilcoxon’s rank sum test. The *P*-values are presented as follows: ***, *P* < 0.001; **, *P* < 0.01; *, *P* < 0.05. ALT, alanine aminotransferase.
